# Exploring the historical stigma of spironolactone use in breast cancer survivors with alopecia

**DOI:** 10.1097/JW9.0000000000000083

**Published:** 2023-04-18

**Authors:** Michael G. Buontempo, Lina Alhanshali, Jerry Shapiro, Kristen Lo Sicco

**Affiliations:** a Department of Dermatology, Hackensack Meridian School of Medicine, Nutley, New Jersey; b Department of Dermatology, SUNY Downstate College of Medicine, Brooklyn, New York; c The Ronald O. Perelman Department of Dermatology, NYU Grossman School of Medicine, New York, New York

**Keywords:** alopecia, breast cancer, hair loss, spironolactone

**What is known about this subject in regard to women and their families?**

- Spironolactone is a medication commonly used off-label to treat androgenetic alopecia in women.
- There have long been concerns regarding the theoretical risk of the drug having the potential to increase the risk of breast cancer due to its antiandrogenetic effects.
- A case series reported in 1975 of 5 patients who developed breast cancer while on spironolactone was later proved to be statistically insignificant.
- In the early 1980s, studies in Japan and America showed that rats given potassium canrenoate, a metabolite of spironolactone, at doses greater than 100 times those given to humans, had an increase in mammary tumors.
- Further molecular studies in 1988 showed that potassium canrenoate produces mutagenic epoxy-canrenone derivatives, which are not produced by spironolactone.
- Spironolactone was subsequently reintroduced into clinical use after these studies cleared it of any association with breast cancer.
- Despite this, the drug has continued to be stigmatized and underutilized, and some patients are wary of using it as a therapeutic agent for their alopecia.

**What is new from this article as messages for women and their families?**

- A 2022 large systematic review and meta-analysis by Bommaredy et al. has confirmed the absence of any association between spironolactone use and breast cancer risk.
- The Food and Drug Administration no longer lists “carcinoma of the breast” as a possible adverse effect of spironolactone since the 2018 spironolactone drug label revisions.
- Women with breast cancer may develop alopecia through several mechanisms, and spironolactone can be an effective treatment option.
- The historical association between breast cancer and spironolactone is unfounded, and women with breast cancer should not be discouraged from using spironolactone for their alopecia treatment.

Spironolactone is an antihypertensive medication that is commonly used off-label to treat androgenetic alopecia (AGA) in women. However, due to its antiandrogenetic effects, there have long been concerns regarding a theorized risk of the drug having the potential to increase the risk of breast cancer. Since the 1980s, this association has been rebutted with molecular studies from 1988 by Cook et al.^1^ and Oppermann et al^2^. and recently as well by a 2022 large systematic review and meta-analysis by Bommaredy et al.^3,4^ Here, we review the historical background, assess why there has long been a perceived association between spironolactone use and breast cancer risk without evidence, and advocate for the role of spironolactone in the management of various alopecias in female breast cancer survivors.

In 1975, a case series reported 5 patients who developed breast cancer while on spironolactone, but Jick and Armstrong^5^ later proved the association to be statistically insignificant. In the early 1980s, studies in Japan, followed by further testing in America, showed that rats given potassium canrenoate, a metabolite of spironolactone, at doses greater than 100 times those given to humans, had an increase in mammary tumors.^6^ These studies prompted a bulletin release calling for the withdrawal of spironolactone in the U.K.

However, further molecular studies in 1988 showed that potassium canrenoate produces mutagenic epoxy-canrenone derivatives, which are not produced by spironolactone.^1^ Spironolactone was subsequently reintroduced into clinical use after these studies cleared it of any association with breast cancer. Despite this, the drug has continued to be stigmatized and underutilized. Since the 2018 spironolactone drug label revisions, the FDA no longer lists “carcinoma of the breast” as a possible adverse effect of spironolactone. This adverse effect was previously listed without a cause-and-effect relationship having been established.

The historical association between breast cancer and spironolactone has led to some patients being wary of using spironolactone as a therapeutic agent for their alopecia. This unfounded association with breast cancer prevents women from receiving important alopecia treatment when they need it most, increasing the psychological burden these patients endure. Up to 14% of patients with cancer may consider rejecting curative cancer treatments if they are associated with hair loss.^7^

Women with breast cancer are more likely to suffer from alopecia through several mechanisms. Depending on their cancer therapy, they may develop anagen effluvium, AGA unmasked by telogen effluvium, or endocrine-induced alopecia, all of which can improve with spironolactone.^7^ Perceived negative associations with spironolactone may discourage some physicians from offering it as a treatment option for breast cancer survivors with AGA and endocrine-induced alopecia. Additionally, patients who learn about this historical association of spironolactone with increased breast cancer risk may have reservations regarding its use.

In conclusion, this article aims to underscore the importance of considering the use of spironolactone for androgenetic and hormone-related alopecia in breast cancer patients without fear of increasing the risk of cancer recurrence.

## Conflicts of interest

JS is a consultant for Lilly, Pfizer, and Replicel Life Sciences. Drs. JS and KLS have been investigators for Regen Lab and are investigators for Pfizer. KLS is a consultant for Pfizer and Aquis. Other authors have no conflicts to disclose.

## Funding

None.

## Study approval

N/A

## Author contributions

All authors contributed to the design of the study. MGB and LA conducted the literature review and wrote the manuscript. All authors contributed to data analysis and interpretation. J.S. and K.L. provided critical revisions to the manuscript. All authors approved the final version of the manuscript for submission.

## Prior presentation

This article has not been previously published and is not under consideration for publication elsewhere.

## Data availability

There is no associated data being stored related to this study.
